# Environmental Toxic Metals and Metalloids Exposure and Cardiovascular Health: Current Evidence from Oxidative Stress and Inflammatory Biomarkers as Indicators of Subclinical Cardiovascular Effects

**DOI:** 10.3390/antiox15091104

**Published:** 2026-09-01

**Authors:** Silvia Baldacci, Elisa Bustaffa, Olivia Curzio, Andrea Borghini, Gabriele Donzelli, Chiara Cavigli, Fabrizio Minichilli

**Affiliations:** Institute of Clinical Physiology, National Research Council, 56124 Pisa, Italy; elisa.bustaffa@cnr.it (E.B.); olivia.curzio@cnr.it (O.C.); andrea.borghini@cnr.it (A.B.); gabrieledonzelli@cnr.it (G.D.); chiaracavigli@cnr.it (C.C.)

**Keywords:** toxic metals, metalloids, cardiovascular disease, oxidative stress, inflammation, biomarkers, environmental exposure, cardiovascular risk

## Abstract

Cardiovascular diseases remain the leading cause of global mortality, and environmental toxic metals and metalloids (TMMs) exposure has emerged as an important and potentially modifiable cardiovascular (CV) risk factor. Due to their persistence, bioaccumulation, and widespread distribution, represent a significant public health concern and have been associated with adverse CV outcomes. Mechanistically, TMM-induced cardiotoxicity is primarily mediated through oxidative stress (OS) and inflammatory pathways. Accordingly, related biomarkers, may be considered markers of biological effect and potential pathophysiological mediators associated with TMM exposure. This narrative review summarizes epidemiological evidence linking environmental TMMs exposure with OS and inflammatory biomarkers to CV outcomes. A structured literature search identified 1915 records in PubMed, of which 42 epidemiological studies met the inclusion criteria. The reviewed evidence consistently indicates that environmental and occupational TMMs exposure is associated with significant alterations in biomarkers of lipid peroxidation, antioxidant defense, systemic inflammation, and endothelial dysfunction, all of which reflect early biological CV effects associated with exposure. However, substantial heterogeneity across studies and the predominance of cross-sectional designs limit causal inference and the evaluation of their predictive capacity for future CV events. Future prospective studies integrating mixture-based exposure assessment, standardized biomarker measurements, and multi-omics approaches are needed to clarify whether these biomarkers can serve as markers of biological effect or subclinical CV toxicity and as potential mediators of CV risk, while also assessing their potential contribution to cardiovascular risk stratification in exposed populations.

## 1. Introduction

Cardiovascular diseases (CVDs) are the leading cause of morbidity and mortality worldwide, accounting for millions of deaths annually and representing a major global public health challenge [1,2]. Their etiology is multifactorial, resulting from complex interactions between genetic background, sociodemographic factors, lifestyle habits, and environmental exposures. Smoking, alcohol consumption, sedentary behavior, and an unhealthy diet are well-known modifiable CVD risk factors [3,4,5,6]. In recent years, environmental exposures, particularly to TMMs, have received increasing attention as potentially modifiable contributors to CV risk [7,8].

Over the past decades, a growing body of epidemiological and toxicological evidence highlighted TMMs such as arsenic (As), cadmium (Cd), lead (Pb), and mercury (Hg) as relevant CV toxicants capable of disrupting CV homeostasis [9,10,11,12,13]. Recently, the American Heart Association identified these toxic metals as a modifiable CV risk factor [14].

TMMs are naturally occurring elements in the Earth’s crust that can exert substantial adverse effects on human health [15]. In CV research, these elements are typically categorized into non-essential toxic elements and essential trace elements. Non-essential elements—including toxic metals such as Pb, Cd, and Hg, as well as the metalloid As, have no physiological role and may exert adverse Cv effects even at low exposure levels [16]. Conversely, essential trace elements (e.g., copper (Cu), chromium (Cr), cobalt (Co), nickel (Ni), and manganese (Mn)) are required in trace amounts for biological functions. However, at excessive levels or in highly toxic chemical species (such as hexavalent chromium), these essential elements can also trigger adverse CV effects and act as recognized CV risk factors [12,17]. Thus, both non-essential toxic elements and excessive exposure to essential elements may contribute to CV toxicity.

Rapid industrialization, urbanization, increased vehicular traffic, intensive agricultural activities, and metal-processing industries have contributed substantially to environmental metal contamination worldwide. Because these pollutants are persistent and non-biodegradable, they accumulate in air, soil, and water and can biomagnify throughout the food chain, resulting in chronic human exposure [18,19]. Human exposure occurs primarily through inhalation of contaminated air, ingestion of contaminated food and water, and dermal contact in both occupational and environmental settings [20,21]. Consequently, TMMs exposure represents a significant CV health concern for both occupationally exposed workers and populations living in contaminated environments.

Human biomonitoring has become an essential tool for assessing internal exposure to TMMs in epidemiological studies. Biological matrices such as blood, urine, hair, and saliva provide integrated measures of exposure from multiple sources and pathways [22]. In recent years, research has increasingly focused on molecular and biochemical biomarkers of effect to better characterize the biological impact of exposure and clarify biological pathways linking TMMs exposure to adverse health outcomes [23].

Mechanistically, TMMs trigger interconnected pathways involving OS, inflammation, and cellular dysfunction. These processes contribute to endothelial injury, vascular remodeling, and the establishment of a chronic pro-inflammatory state, thereby promoting the development and progression of atherosclerosis, hypertension, and other CVDs [11,12,17,24,25]. Persistent disruption of vascular homeostasis may increase the long-term risk of major adverse CV events, including myocardial infarction (MI) and stroke.

In this context, biomarkers of OS and inflammation emerged as potential markers of early biological effects associated with metal exposure. Growing epidemiological evidence suggests that alterations in OS and inflammatory biomarkers may reflect biological responses to TMMs exposure in both general and occupational populations [26,27,28]. These biomarkers may therefore provide useful information on early biological responses to TMMs exposure and may contribute to elucidating potential pathophysiological pathways linking environmental metal exposure with cardiovascular effects. However, the predictive value of OS and inflammatory biomarkers for future CVD events remains insufficiently established and requires confirmation in large prospective studies before these biomarkers can be considered reliable tools for CV risk prediction [29,30,31]. The available evidence remains fragmented, highlighting the need for well-designed longitudinal studies to clarify their prognostic utility.

Therefore, the aim of this review is to summarize current epidemiological evidence on the association between environmental exposure to TMMs (i.e., As, Pb, Cd, Hg, Co, Cr, Cu, Mn, and Ni) and CVD-related biomarkers, with particular emphasis on OS and inflammation as key mechanisms underlying metal-induced CV toxicity.

## 2. Materials and Methods

### 2.1. Study Design and Search Strategy

This narrative review was conducted using a structured literature search approach to identify recent epidemiological evidence on associations between TMMs exposure, OS and inflammatory biomarkers, and CV outcomes. Although this is a narrative review, a structured literature search and predefined eligibility criteria were adopted to enhance transparency and reproducibility.

A comprehensive literature search was conducted in the PubMed database to identify relevant epidemiological studies published in the last 10 years (up to December 2025) without language restriction. The search was limited to studies involving human participants and publications with full-text availability. PubMed was selected because it provides comprehensive coverage of biomedical and epidemiological literature relevant to the objectives of this review.

The search strategy combined MeSH terms and keywords related to TMMs exposure, CV outcomes, and biomarkers of OS and inflammation. The following search string was used:

[(Heavy metal OR “Arsenic”[MeSH Terms] OR “Lead”[MeSH Terms] OR “Pb” OR “chromium”[MeSH Terms] OR “cobalt”[MeSH Terms] OR “mercury”[MeSH Terms] OR “Cadmium”[MeSH Terms] OR “nickel”[MeSH Terms] OR “manganese”[MeSH Terms] OR “copper”[MeSH Terms]) AND (“cardiovascular risk”[Title/Abstract] OR “cardiovascular disease”[Title/Abstract] OR “cardiovascular mortality”[Title/Abstract] OR “myocardial infarction”[Title/Abstract] OR “myocardial ischemia”[Title/Abstract] OR “coronary heart disease”[Title/Abstract] OR cardiovascular abnormalities[Title/Abstract] OR “ischemic”[Title/Abstract] OR “stroke”[Title/Abstract] OR “cerebrovascular”[Title/Abstract] OR “CHD” [Title/Abstract] OR “CVD” [Title/Abstract] OR “heart diseases”[Title/Abstract] OR “Hypertension”[Title/Abstract] OR Hypertenive[Title/Abstract] OR “blood pressure”[Title/Abstract]) AND (exposure OR oxidative stress OR inflammation OR adverse outcome pathways OR “early biological effect” OR biomarkers)]. Boolean operators (AND/OR) were used to combine search terms.

**Figure 1:**
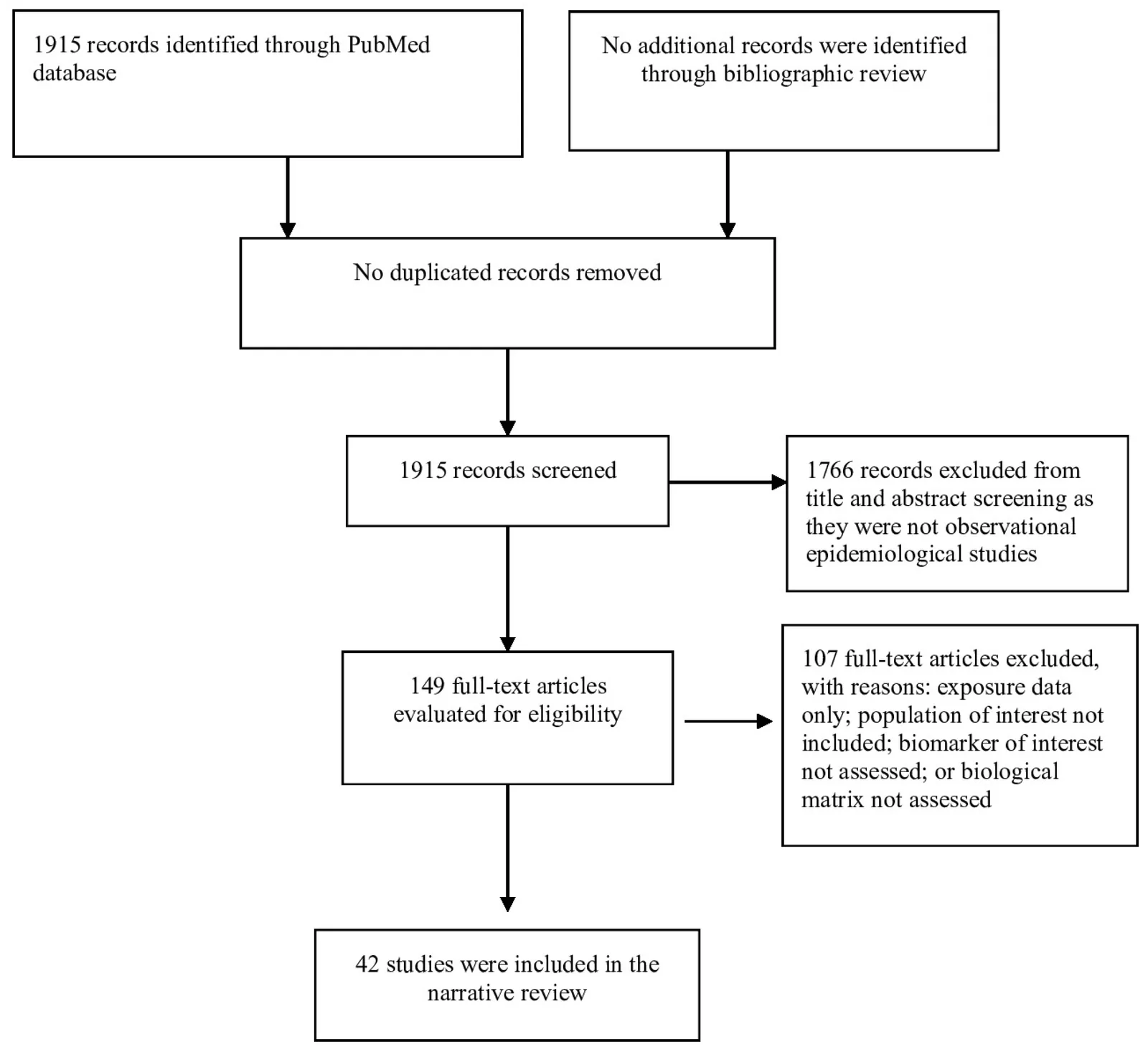
Flowchart illustrating studies selection process.

The search yielded 1915 records. Among all identified records, 1766 studies were excluded after title/abstract screening, and 149 full texts of potentially eligible articles were assessed for inclusion. Ultimately, 42 studies meeting the eligibility criteria were included in the review (Figure 1).

### 2.2. Data Extraction

For synthesis, included studies were grouped according to the main mechanistic pathway: OS and inflammation. For each study, data were systematically extracted and tabulated, including first author, year of publication, country, study design, sample size, data source, exposure assessment methods (biological matrices and analytical techniques), biomarkers of effect, CV outcomes assessed, and main findings.

### 2.3. Inclusion and Exclusion Criteria

Eligible studies were observational epidemiological studies (cross-sectional, case–control, or cohort) conducted in general populations or occupational cohorts. Studies were included if they assessed TMMs exposure using validated biological matrices (e.g., blood, urine, toenails, or other biomarkers of internal dose) and reported associations with at least one OS and/or inflammatory biomarker and CV outcomes or risk indicators.

OS biomarkers included lipid peroxidation markers (malondialdehyde (MDA), thiobarbituric acid reactive substances (TBARSs), 8-isoprostanes, F2-isoprostanes (F2-IsoPs), 8-iso-prostaglandin F2α (8-iso-PGF2α), oxidative DNA damage markers 8-hydroxy-2′-deoxyguanosine (8-OHdG), antioxidant enzymes (superoxide dismutase (SOD), glutathione peroxidase (GPx), and related redox biomarkers (oxidized low-density lipoprotein), glutathione (GSH)-related markers).

Inflammatory biomarkers included cytokines (interleukins (IL): IL-1β, IL-6, IL-8, IL-10, IL-18), tumor necrosis factor-α (TNF-α), C-reactive protein (CRP), high-sensitivity CRP (hs-CRP), monocyte chemoattractant protein-1 (MCP-1), myeloperoxidase, plasminogen activator inhibitor-1 (PAI-1), serum amyloid A, fibrinogen, vascular cell adhesion molecule-1 (VCAM-1), and intercellular adhesion molecule-1 (ICAM-1).

Studies were excluded if they were non-original articles (reviews, editorials, letters, comments, book chapters), experimental in vitro or in vivo studies, or if they relied solely on exposure assessment without biological matrices (e.g., self-reported exposure). Studies focusing exclusively on pregnant women, children, or adolescents were also excluded.

## 3. Results

### 3.1. Overview of Oxidative Stress in Toxic Metal and Metalloid Exposure

OS is a key mechanism underlying TMM-induced CV toxicity. Increased reactive oxygen species (ROS) production and impaired antioxidant defenses contribute to endothelial dysfunction, vascular remodeling, lipid peroxidation, mitochondrial damage, and inflammation, thereby promoting atherosclerosis, hypertension, and other CVDs [32,33,34,35]. TMMs induce OS primarily through enhanced ROS generation and depletion of antioxidant systems.

This review focused on lipid peroxidation biomarkers (MDA, TBARS, 8-isoprostanes, F2-IsoPs, 8-isoPGF2α), oxidative DNA damage marker 8-OHdG, and antioxidant enzymes (SOD, GSH, glutathione peroxidase). Included observational studies are summarized in Table 1.

#### 3.1.1. Lipid Peroxidation and Antioxidant Defense Biomarkers

A community-based case–control study conducted in India reported higher hair and nail concentrations of Cd, Pb, and As levels in hypertensive subjects, accompanied by increased TBARS and protein carbonyl levels, along with reduced SOD, GSH levels, and total antioxidant capacity [39]. Similarly, in a cross-sectional study, the exposure to Co, Ni, and Pb, in a Chinese e-waste area, was associated with elevated MDA and 8-isoprostane levels, with Ni showing the strongest association with CV biomarkers [40]. Residents near an electric arc furnace in Taiwan showed higher Pb exposure, increased MDA, and reduced GSH levels [42].

In the New Hampshire Health Study population-based U.S. study, urinary As was associated with increased 15-F2t-isoprostane, while methylation efficiency was inversely related to OS [44].

Occupational studies in India and China further showed that Pb exposure in battery workers was linked to hypertension, elevated MDA, and reduced antioxidant defenses including GSH, GST and SOD biomarkers respectively [41,43]. Ko et al. reported that welding-related exposure, particularly to Cr and Mn, was also associated with increased MDA levels [45].

A Chinese cohort using Bayesian kernel machine regression (BKMR) found that combined exposure to Mn, Cr, and Mo was associated with higher urinary 8-isoPGF2α and blood pressure, with mediation by OS, whereas for 8-OHdG, no significant association was observed [36].

#### 3.1.2. Oxidative DNA Damage Biomarkers (8-OHdG)

In an occupational cross-sectional study of 199 Bangladeshi e-waste workers and 104 controls, blood Pb was significantly associated with elevated systolic blood pressure, whereas urinary 8-OHdG levels were unexpectedly lower in exposed workers and showed no mediating effect [37]. The authors suggested that the lack of association between metal exposure and urinary 8-OHdG may partly reflect a relatively low biologically relevant dose in the study population. They also noted that lifestyle, dietary patterns, nutritional status, physiological factors, socioeconomic status, and other environmental exposures may influence urinary 8-OHdG levels. In contrast, a hospital-based case–control study of 110 MI patients and 110 controls exposed to industrial pollution in India demonstrated that elevated hair concentrations of toxic metals (Pb, As) positively correlated with serum 8-OHdG levels and adverse CV profiles, whereas essential trace elements (Cu, Mn) were inversely associated with oxidative DNA damage [38]. These contrasting findings may partly reflect differences in exposure dose, exposure context, biological matrix (urinary vs. serum) and underlying population health status healthy occupational workers vs. acute clinical MI cases). Furthermore, the absence of a mediation effect in the Bangladeshi study may indicate that the associations between TMMs and hematological or CV outcomes operate through mechanisms other than the 8-OHdG pathway or may reflect the low level of the mediating biomarker and the complexity of the underlying biological mechanisms [37]. These contrasting findings illustrate how population characteristics, biological matrices (urinary vs. serum 8-OHdG), and specific metal toxicodynamics contribute to the heterogeneity of 8-OHdG responses across studies.

### 3.2. Overview of Inflammatory Mechanisms in Toxic Metals and Metalloids Exposure

Chronic inflammation is a key mechanism in the development and progression of CVDs. Endothelial injury, OS, lipid accumulation, and persistent immune activation contribute to vascular dysfunction, atherosclerotic plaque formation, and thrombotic events [46,47,48]. TMMs may exacerbate these processes through activation of inflammatory signaling pathways and disruption of vascular homeostasis.

Inflammatory biomarkers include CRP and hs-CRP, cytokines (IL-6, TNF-α), chemokines (MCP-1), endothelial markers (VCAM-1, ICAM-1), coagulation factors (fibrinogen, PAI-1), and acute-phase proteins (serum amyloid A). Included observational studies are summarized in Table 2.

#### 3.2.1. CRP and hs-CRP Biomarkers

Large population-based studies consistently link TMMs exposure with elevated CRP/hs-CRP levels. In the All of Us Research Program (USA), BKMR analyses demonstrated that Cd showed the strongest positive association with CRP, while As and Hg were more strongly related to lipid and blood pressure profiles [49]. Similarly, multiple National Health and Nutrition Examination Survey (NHANES)-based analyses demonstrated that Cd exposure was associated with elevated CRP and increased CVD risk [66], including coronary heart disease, heart failure, and stroke [58,62]. Furthermore, Cd, Pb, and Hg were associated with increased CVD mortality, with evidence suggesting partial mediation through CRP [51].

Consistent findings were observed in Korean National Health and Nutrition Examination Survey (KNHANES) data, where Cd and Pb exposure were associated with adverse CV profiles and elevated CRP, while Hg exposure was specifically associated with increased hs-CRP [56,65]. In Iran, the Mashhad Stroke and Heart Atherosclerotic Disorder Study reported higher hs-CRP levels in hypertensive individuals and identified associations between Cu imbalance and hypertension, supporting a role for metal dysregulation in inflammatory CV risk [69].

Occupational and environmental studies support these findings. In a highly metal-exposed industrial population in India, Pb and As exposure was positively correlated with hs-CRP in MI patients [38]. Similarly, NHANES occupational analyses confirmed associations between Pb exposure, elevated hs-CRP, and increased blood pressure [74].

Disease-specific evidence further indicates that metal exposure is linked to inflammatory activation. Elevated Cu and other trace metals were associated with increased MI risk and severity, partly mediated by hs-CRP [52], while Cu and Cu/Zinc (Zn) imbalance were also linked to heart failure and systemic inflammation [59]. Elevated toenail Cu levels were also reported in intracerebral hemorrhage and hypertension patients, with hs-CRP correlating with disease severity [72]. As exposure was associated with MI severity and inflammatory markers [57], and mixed metal exposure (Pb, Cd, Hg, As) was related to stroke severity, with Hg and As positively correlated with CRP [63]. In addition, Cr deficiency has been associated with hypertension and elevated hs-CRP in diabetic populations [73].

Prospective and cohort studies further support these associations. In a Finnish cohort study, an elevated Cu/Zn ratio was associated with an increased risk of heart failure, with a dose–response relationship observed for Cu exposure. In addition, Cu/Zn ratio was positively correlated with hs-CRP [61]. Similarly, a cross-sectional study reported positive associations among plasma Cu concentrations, CRP levels, and coronary heart disease risk [68]. Malmö Diet and Cancer Study found that higher Cd exposure was associated with elevated hs-CRP and increased risk of CV events [80]. However, NHANES-based longitudinal data have shown that blood Pb levels remain associated with CVD mortality after adjustment for serum CRP and other potential confounders [79].

#### 3.2.2. Multi-Protein Inflammatory and Endothelial Biomarkers

Evidence from population-based studies suggests that TMMs exposure is associated with multi-protein inflammatory, endothelial, and coagulation pathways beyond CRP. In a Swedish community-based study of contaminated glassworks areas, long-term As and Pb exposure was associated with increased CVD and stroke risk, along with elevated inflammatory mediators such as MCP-1, macrophage inflammatory protein-1 alpha, IL-12/IL-23p40, vascular endothelial growth factor-C (VEGF-C), IL-1α, and VEGF, indicating activation of multiple inflammatory pathways [50]. In Taiwan, among residents living near an electric arc furnace, Pb exposure was linked to increased blood pressure but not to inflammatory markers including IL-6 and IL-8 [42]. In China, metal mixtures (Cr, Cu, Cd, Pb) were associated with higher Framingham Risk Scores and endothelial dysfunction markers (VCAM-1, ICAM-1), with urinary Cu and Cd showing positive associations with VCAM-1 [54].

In Finland, higher blood Cu was associated with increased hs-CRP and CV risk but not with PAI-1, suggesting a stronger link to systemic inflammation than fibrinolysis [70]. In Sweden, Cd exposure was associated with soluble urokinase plasminogen activator receptor (suPAR) but not with CRP [75], while in Spain Cd exposure was linked to higher growth differentiation factor-15 levels, partially mediated by IL-6 and hs-CRP [60]. In the U.S., based on New Hampshire Health Study data, As exposure was associated with VCAM-1 but not TNF-α or ICAM-1, indicating selective endothelial activation [44].

Occupational studies further highlight heterogeneous inflammatory responses. Dust storm-exposed workers showed associations between As, Cd, and Cr with increased blood pressure, fibrinogen, and TNF-α but decreased CRP, suggesting complex immune regulation [55]. In Swedish hard metal workers, Co exposure was associated with increased IL-6 and weaker associations with TNF-α, IL-8, IL-10, and CC16 [64]. Among traffic enforcers in Manila, blood Pb was positively associated with hs-CRP but not with sICAM-1 or soluble VCAM-1 (sVCAM-1) [53]. Similarly, occupational Pb exposure has been shown to promote atherosclerosis and CVD development by altering inflammatory and coagulation-related markers such as CRP and fibrinogen, particularly among highly exposed workers [77].

Clinical evidence supports multi-pathway involvement. In acute ischemic stroke patients, elevated Cu, Mn, As, Pb, and Ni were associated with fibrinogen, D-dimer, complement factors, and VEGF, consistent with inflammatory, coagulation, and endothelial injury mechanisms [78].

Prospective findings are not entirely consistent. In Kosovo young adults, Pb exposure was associated with higher blood pressure and endothelial dysfunction markers (sVCAM-1, sICAM-1) [71]. The Strong Heart Study reported associations between urinary As and fibrinogen but not hs-CRP, indicating selective coagulation effects [76]. However, a longitudinal study found no associations between urinary As, Cd, or Hg and circulating CRP, IL-6, TGF-β1, ET-1, or VEGF [36], while the Multi-Ethnic Study of Atherosclerosis observed no associations between urinary As concentrations and hs-CRP, IL-6, fibrinogen, vascular dysfunction, or subclinical atherosclerosis [67], highlighting variability across populations and exposure contexts.

## 4. Discussion

**Figure 2:**
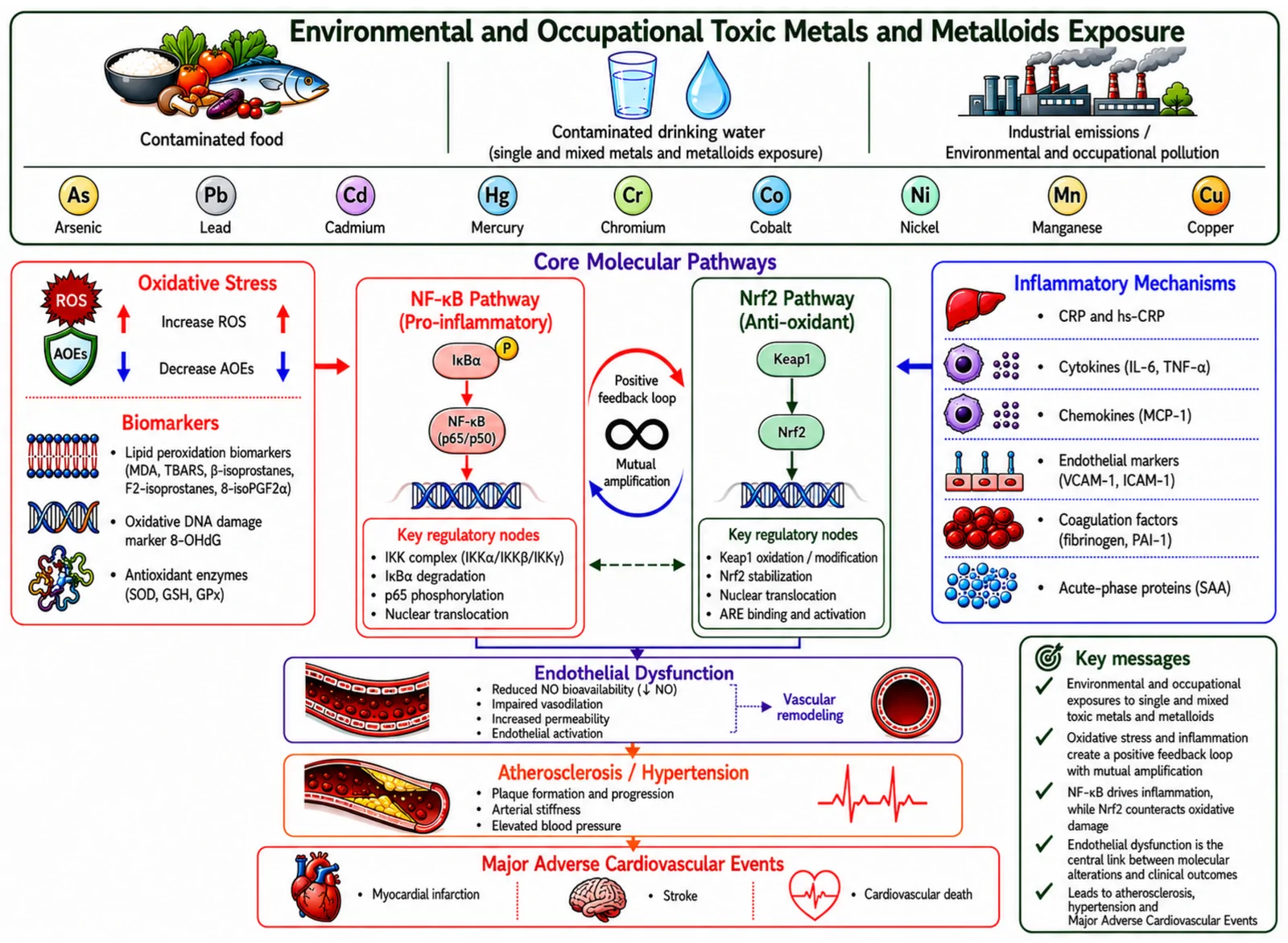
Overview of toxic metal and metalloid-induced molecular mechanisms potentially contributing to cardiovascular diseases (CVDs), including oxidative stress (OS) and chronic inflammation pathways with related biomarkers. **Abbreviations:** AOEs, antioxidant enzymes; ARE, antioxidant response element; As, arsenic; Cd, cadmium; Co, cobalt; CRP, C-reactive protein; Cr, chromium; Cu, copper; DNA, deoxyribonucleic acid; GPx, glutathione peroxidase; GSH, glutathione; Hg, mercury; hs-CRP, high-sensitivity C-reactive protein; ICAM-1, intercellular adhesion molecule-1; IκBα, Inhibitor of Nuclear Factor Kappa B alpha; IκB, Inhibitor of κB; IKK complex, IκB kinase complex; IKKα, IκB kinase alpha; IKKβ, IκB Kinase beta; IκB, inhibitor of κB; IKKγ, IκB kinase gamma; IL-6, interleukin-6; Keap1, Kelch-like ECH-associated protein 1; MDA, malondialdehyde; MCP-1, monocyte chemoattractant protein-1; Mn, manganese; NF-κB, nuclear factor kappa B; Ni, nickel; NO, nitric oxide; Nrf2, nuclear factor erythroid 2-related factor 2; 8-OHdG, 8-hydroxy-2′-deoxyguanosine; PAI-1, plasminogen activator inhibitor-1; Pb, lead; ROS, reactive oxygen species; SAA, serum amyloid A; SOD, superoxide dismutase; TBARS, thiobarbituric acid-reactive substances; TNF-α, tumor necrosis factor-alpha; VCAM-1, vascular cell adhesion molecule-1; 8-iso-PGF2α, 8-isoprostaglandin F2α.

The present narrative review summarizes current epidemiological evidence evaluating potential associations between environmental and occupational TMMs exposure and CV outcomes, with a particular focus on OS and inflammatory biomarkers as markers of biological effect. Across diverse study designs, including population-based cohorts, occupational investigations, and clinical studies, exposure to Pb, Cd, As, Cr, Ni, Co, Mn, Hg, and Cu was associated with adverse CV outcomes in some settings. Although the strength of associations varied across populations and exposure scenarios, and while the predominance of cross-sectional data limits definitive causal conclusions, the available evidence suggests that TMMs may contribute to CV risk through plausible pathophysiological pathways, including disturbances in redox homeostasis, endothelial dysfunction, mitochondrial impairment, and chronic inflammatory activation (Figure 2), rather than through a simple deterministic linear cascade.

### 4.1. Oxidative Stress Pathways and Related Oxidative Stress Biomarkers

The evidence reviewed suggests that OS represents a central mechanism linking TMMs exposure to CV toxicity. However, the current body of epidemiological evidence remains limited, as most available studies are cross-sectional or case–control in design and therefore unable to establish temporal or causal relationships. Furthermore, substantial heterogeneity in exposure assessment, biomarker selection, analytical methods, and population characteristics complicates direct comparisons across studies.

Despite these limitations, biomarkers of lipid peroxidation and antioxidant defense appear relatively consistent associations with TMM exposure and adverse CV exposure and adverse CV profiles. Elevated concentrations of MDA, TBARS, 8-isoprostanes, and 8-isoPGF2α were repeatedly observed among individuals exposed to non-essential TMMs, including Pb, Cd, As, and Hg. Similar alterations have also been observed following excessive exposure to essential trace and transition elements, such as Cr, Ni, Cu, Mn, and Co. However, important biological and etiological differences should be considered. Whereas non-essential elements have no established physiological role and may exert toxic effects even at relatively low exposure levels, essential trace elements are tightly regulated by homeostatic mechanisms, and both deficiency and excessive exposure can disrupt redox homeostasis and adversely affect CV health. Among these biomarkers, F2-IsoPs and 8-isoPGF2α are generally considered relatively specific and stable markers of lipid peroxidation and may provide a more informative assessment of systemic OS compared with MDA and TBARS.

Concurrently, several studies reported reduced levels of endogenous antioxidant defenses, including SOD, GSH, GST, and total antioxidant capacity. These findings are biologically plausible, as many toxic metals promote excessive ROS generation while directly inhibiting antioxidant enzymes or increasing antioxidant consumption. The resulting imbalance between oxidant production and antioxidant protection may contribute to endothelial injury, lipid oxidation, vascular remodeling, and progression of atherosclerotic disease [11,12,81].

In contrast, evidence regarding oxidative DNA damage, primarily assessed through 8-OHdG, was less consistent. This heterogeneity may be explained by differences in population characteristics, exposure intensity and duration, exposure patterns, and analytical methods. Occupational cohorts (e.g., e-waste workers) [37] and healthy young adults [36] showed non-significant or even lower 8-OHdG levels, whereas stronger positive associations were observed in clinical populations with established CV disease, such as MI patients [38]. Similarly, elevated 8-OHdG levels were observed in MI patients with higher Pb and As exposure, while studies of Cr, Mn, Pb, and Cd reported no significant associations after adjustment for potential confounders. Differences in exposure intensity and duration, acute versus chronic exposure, biomarker kinetics and renal excretion, underlying susceptibility and disease status, and analytical methodology may contribute to these discrepancies. In addition, residual confounding by factors such as age, smoking, and renal function cannot be excluded. Overall, these findings suggest that 8-OHdG should be interpreted in relation to exposure characteristics, population health status, and analytical methodology rather than as a uniform marker of metal-related oxidative DNA damage.

In this context, comprehensive consideration of major potential confounding factors is essential for accurate risk interpretation. Renal function represents a critical biological confounder, as variations in glomerular filtration and renal clearance directly alter the excretion kinetics and systemic concentrations of both toxic metals and circulating biomarkers. Concurrently, socioeconomic status serves as a broad structural confounder that profoundly influences dietary habits, place of residence, housing conditions, lifestyle behaviors (such as smoking), and occupational exposure profiles. Because these physiological and sociodemographic factors can simultaneously influence exposure levels and underlying CV risk, failing to adequately adjust for them may distort observed correlations. Future prospective studies must therefore incorporate systematic adjustments for renal function metrics and comprehensive socioeconomic indicators to isolate the independent CV effects of metal exposure.

Overall, the available evidence indicates that biomarkers of lipid peroxidation and antioxidant depletion may serve as markers of biological effect reflecting early cardiovascular responses of early biological CV effects associated with TMMs exposure. Nevertheless, their utility as predictors of future CV disease remains insufficiently established. Because most studies evaluated biomarker alterations and CV outcomes simultaneously, it remains unclear whether these biomarkers precede disease development or simply reflect ongoing pathological processes. Large prospective studies incorporating repeated exposure assessment, standardized biomarker measurements, and long-term CV follow-up are therefore needed to determine their prognostic value and potential application in environmental CV risk assessment.

### 4.2. Inflammatory Pathways and Related Biomarkers

Available evidence indicates that systemic inflammation represents a key mechanism through which TMMs exposure contributes to CVD. Alterations in a broad range of inflammatory and vascular biomarkers have been reported in association with exposure to cardiotoxic metals, supporting the involvement of chronic low-grade inflammation in TMM-induced CV toxicity.

Among the inflammatory biomarkers investigated, CRP and hs-CRP showed the most consistent associations with both TMMs exposure and adverse CV outcomes. Evidence from large population-based studies, occupational investigations, clinical studies, and cohort analyses indicates that exposure to Cd, Pb, As, and Hg is frequently associated with elevated CRP and hs-CRP concentrations. These biomarkers are well-established mediators of systemic inflammation and clinically relevant predictors of CV risk. Hs-CRP has emerged as a robust predictor of future CV events and may provide prognostic information beyond traditional CV risk factors [82,83,84].

Large epidemiological studies, including NHANES, KNHANES, and the All of Us Research Program, consistently reported positive associations between Cd exposure and increased CRP or hs-CRP concentrations, which were further linked to a higher risk of coronary heart disease, heart failure, stroke, and CV mortality. Similar relationships were observed for Pb, As, Hg, and Co, although the strength and consistency of these associations varied across populations, exposure levels, and study designs. Importantly, several investigations identified CRP as a partial mediator of the association between metal exposure and CV outcomes, suggesting that systemic inflammation may represent a biologically plausible pathway linking environmental metal exposure to CV damage.

Additional evidence from occupational and clinical studies further supports the role of inflammatory activation in clinically manifest CVD. Positive associations between Cu exposure, increased CRP concentrations, and adverse CV outcomes were observed in both population-based and disease-specific studies. Prospective investigations reporting associations between elevated Cu concentrations, increased Cu/Zn ratios, and future heart failure risk further suggest that disruption of metal homeostasis may contribute to CV pathology through inflammatory mechanisms.

Beyond CRP and hs-CRP, several studies identified alterations in cytokines, chemokines, and endothelial biomarkers that provide additional insight into the pathophysiology of TMM-related CVD. Elevated concentrations of MCP-1, macrophage inflammatory protein-1 alpha, IL-12/IL-23p40, TNF-α, and IL-6 were associated with exposure to As, Pb, Cd, and Co, indicating activation of multiple inflammatory signaling pathways. Biomarkers of endothelial dysfunction, including VCAM-1, ICAM-1, sVCAM-1, and sICAM-1, were also positively associated with exposure to As, Cd, Cu, and Pb in several studies, supporting the hypothesis that endothelial activation represents an early event in metal-induced vascular injury. Because endothelial dysfunction promotes leukocyte adhesion, vascular inflammation, and atherosclerotic plaque formation, these biomarkers may provide mechanistic links between TMMs exposure and CVD progression.

Available evidence also supports the involvement of coagulation, fibrinolytic, and vascular remodeling pathways. Elevated fibrinogen and D-dimer concentrations were observed among individuals exposed to multiple metals, suggesting a prothrombotic state that may increase susceptibility to CV events. Alterations in plasminogen activator inhibitor-1 (PAI-1), VEGF, and VEGF-C further indicate potential effects on fibrinolysis, angiogenesis, and vascular remodeling. In addition, emerging biomarkers such as suPAR and growth differentiation factor-15 were associated with Cd exposure, suggesting that these circulating molecules may serve as sensitive markers of biological effect, reflecting subclinical immune activation, cellular stress, and tissue injury beyond that reflected by CRP alone.

An important consideration when interpreting these findings is that most inflammatory biomarkers currently used in epidemiological studies are not specific to TMMs exposure or CVD. Biomarkers such as CRP, hs-CRP, IL-6, TNF-α, and several adhesion molecules reflect generalized inflammatory responses and may be influenced by numerous factors, including obesity, smoking, dietary habits, physical inactivity, infections, chronic inflammatory disorders, metabolic diseases, medication use, and aging. Likewise, commonly used OS biomarkers such as MDA and TBARS are affected by a wide range of endogenous and exogenous stimuli and therefore lack specificity for metal-induced oxidative damage [85,86]. Consequently, although these biomarkers provide valuable information on biological responses associated with environmental exposure, they should be interpreted as mediators of systemic pathophysiological processes rather than specific signatures of TMM toxicity. Integrating multiple complementary biomarkers together with accurate exposure assessment and longitudinal study designs may therefore improve causal interpretation and strengthen their potential role in CV risk assessment.

Despite the overall consistency of the evidence, interpretation of these findings remains challenging because of substantial heterogeneity across studies, including differences in exposure intensity, metal mixtures, exposure duration, biomarker kinetics, analytical methodologies, and population characteristics. Furthermore, inflammatory biomarkers may reflect various stages of CV pathogenesis, ranging from early endothelial activation and immune dysregulation to advanced vascular injury, thrombosis, and clinically overt disease [87]. Consequently, while CRP and hs-CRP currently appear to be the most robust inflammatory biomarkers associated with TMMs exposure, the predictive value of other inflammatory and endothelial biomarkers remains insufficiently established and requires confirmation in prospective studies.

### 4.3. Interplay Between Oxidative Stress and Inflammation: Implications for Cardiovascular Risk Assessment

Although OS and inflammation were discussed separately in this review, the available evidence suggests that these pathways are closely interconnected and likely act synergistically in mediating TMMs-induced CV toxicity. TMMs can promote excessive production of ROS through mitochondrial dysfunction, redox cycling, and depletion of endogenous antioxidant defenses [88]. Increased ROS generation subsequently activates redox-sensitive signaling pathways, including nuclear factor kappa B (NF-κB) and Mitogen-Activated Protein Kinases, stimulating the production of inflammatory cytokines, adhesion molecules, and acute-phase proteins [87]. Conversely, chronic inflammatory activation may further enhance ROS generation through immune-cell recruitment and activation, creating a self-perpetuating cycle of oxidative damage and inflammation [89].

Several studies included in this review provide epidemiological support for this mechanistic interaction. Notably, studies simultaneously evaluating OS and inflammatory biomarkers reported concurrent alterations in lipid peroxidation markers, antioxidant defenses, inflammatory mediators, and markers of CV effects among TMM-exposed populations [36,38,42,44]. These findings suggest that OS and inflammation are not independent biological responses but rather complementary and mutually reinforcing processes contributing to CV injury.

At the molecular level, several highly conserved signaling pathways appear to coordinate the interaction between OS and inflammation following TMMs exposure. Among these, activation of the NF-κB pathway represents one of the best-characterized mechanisms linking ROS generation to the transcription of pro-inflammatory cytokines, chemokines, adhesion molecules, and other mediators involved in endothelial dysfunction and atherogenesis. Persistent NF-κB activation has been consistently associated with vascular inflammation, plaque progression, and increased CV risk [90].

Conversely, the nuclear factor erythroid 2-related factor 2 (Nrf2) pathway constitutes the principal endogenous defense mechanism against OS. Under physiological conditions, Nrf2 regulates the expression of numerous antioxidants and cytoprotective genes, including those involved in GSH synthesis, detoxification enzymes, and cellular redox homeostasis. Experimental evidence suggests that chronic exposure to TMMs may impair or dysregulate Nrf2 signaling, thereby reducing antioxidant capacity, and increasing susceptibility to oxidative vascular injury [91].

Another pathway receiving increasing attention is the nucleotide-binding oligomerization domain-like receptor family pyrin domain-containing 3 (NLRP3) inflammasome. Excessive ROS production induced by TMMs may promote NLRP3 activation, leading to caspase-1 activation and the maturation of pro-inflammatory cytokines such as IL-1β and IL-18. Growing experimental evidence indicates that NLRP3 activation contributes to chronic vascular inflammation, endothelial dysfunction, and atherosclerotic plaque development, suggesting that this pathway may represent a critical mechanistic bridge between environmental exposure and CVD [92].

Although most epidemiological studies included in this review assessed circulating biomarkers rather than intracellular signaling pathways, the growing integration of molecular epidemiology with mechanistic toxicology suggests that NF-κB, Nrf2, and NLRP3 constitute complementary biological pathways underlying many of the OS and inflammatory alterations observed in environmentally exposed populations. Future human studies incorporating pathway-specific biomarkers together with traditional inflammatory and OS markers may further clarify their contribution to CV risk prediction.

The interaction between these pathways is particularly relevant to CVD because both converge on common downstream mechanisms, including endothelial dysfunction, reduced nitric oxide bioavailability, vascular remodeling, lipid oxidation, and atherosclerotic progression. Consequently, no single biomarker appears sufficient to capture the complexity of TMMs-induced CV toxicity. Instead, multi-biomarker approaches integrating markers of OS, inflammation, endothelial dysfunction, coagulation, and vascular remodeling may provide a more comprehensive assessment of CV risk associated with environmental and occupational metal exposure.

An additional finding emerging from the reviewed literature is the growing importance of mixture-based exposure models. Recent studies using BKMR and mediation analyses demonstrated that combined exposure to multiple metals exerts stronger and more biologically relevant effects than single-metal exposure alone. Furthermore, OS and inflammatory biomarkers were identified as potential mediators of the association between metal mixtures and adverse CV outcomes, particularly hypertension, endothelial dysfunction, and coronary artery disease. These observations are highly relevant because environmental and occupational exposures rarely occur in isolation, and single-metal approaches may underestimate the cumulative CV burden associated with real-world exposure scenarios.

The reviewed evidence also suggests that susceptibility to TMM-induced CV toxicity may vary according to exposure duration, age, sex, nutritional status, genetic susceptibility, and pre-existing cardiometabolic conditions. Occupational studies generally reported greater biomarker alterations than environmental population studies, likely reflecting higher exposure intensity and longer exposure duration. Similarly, stronger associations were frequently observed among patients with MI, stroke, or established CVD, suggesting that OS and inflammatory pathways may contribute not only to disease initiation but also to disease progression and severity.

Overall, current evidence supports a continuum from TMMs exposure to molecular alterations, OS, inflammatory activation, endothelial dysfunction, and ultimately overt CVD. However, because most available studies are cross-sectional, the ability of these biomarkers to predict future CV events remains uncertain. Nevertheless, most currently available biomarkers cannot yet be considered predictive biomarkers of CVD, as prospective evidence demonstrating their incremental prognostic value beyond established CV risk factors remains limited.

Large prospective studies incorporating repeated exposure assessment, longitudinal biomarker measurements, and long-term CV follow-up are required to determine whether integrated biomarker panels can improve CV risk prediction and support environmental health surveillance strategies.

### 4.4. Toxic Metal and Metalloids Mixtures and the Exposome Perspective

An emerging aspect of environmental CV epidemiology concerns the evaluation of combined exposure to multiple TMMs rather than individual contaminants. In real-world settings, environmental and occupational exposures rarely occur in isolation, and individuals are typically exposed simultaneously to complex mixtures of metals that may interact through additive, synergistic, or even antagonistic mechanisms. Consequently, evaluating single metals independently may underestimate the overall CV burden associated with environmental exposure.

Recent epidemiological studies have increasingly adopted mixture-based analytical approaches, including BKMR, Weighted Quantile Sum regression, and Quantile G-Computation, to better characterize the joint effects of multiple metals. Evidence from these studies suggests that combined exposure to metals such as Pb, Cd, As, Cr, Mn, Ni, and Cu is associated with greater alterations in OS biomarkers, endothelial dysfunction, inflammatory activation, and blood pressure than those observed for individual metals alone.

Mainstream statistical approaches for metal mixtures offer complementary strengths and are suited to different analytical objectives. Weighted Quantile Sum (WQS) regression constructs a weighted mixture index to estimate the overall effect of a metal mixture and identify influential components. It is particularly useful when the primary objective is to quantify a cumulative mixture effect, and the component-specific associations are expected to be predominantly in the same direction. However, conventional WQS may require sample splitting for estimation and validation, which can reduce statistical power in small cohorts. Quantile G-Computation similarly estimates the overall effect of a joint increase in mixture components but allows component-specific associations to operate in different directions, making it particularly suitable for mixtures in which metals may have heterogeneous or opposing effects. Unlike conventional WQS, Quantile G-Computation similarly does not require sample splitting and provides an interpretable estimate of the overall mixture effect. Conversely, BKMR provides a flexible, non-parametric approach capable of capturing complex non-linear exposure–response relationships, non-additive interactions, and potential threshold effects, although it generally requires greater computational resources. Thus, WQS and Quantile G-Computation are particularly useful for estimating overall mixture effects, whereas BKMR is better suited to investigating complex non-linear relationships and interactions among mixture components.

Crucially, the CV toxicity of metal mixtures depends on the specific interaction patterns—additive, synergistic, or antagonistic—of individual metal combinations. Co-exposure to Pb and Cd has been associated with greater endothelial dysfunction, vascular OS, and blood pressure alterations than exposure to either metal alone, suggesting potential synergistic effects. Similarly, combining As + Cu and As + Cd exposure has been associated with OS and vascular injury, whereas essential elements such as Zn and selenium may attenuate Cd- or Pb-related toxicity through antioxidant and competitive mechanisms. However, the magnitude and direction of these effects may vary according to exposure level, duration, population characteristics, and susceptibility.

Different statistical approaches provide complementary information on mixture effects. WQS is useful for estimating an overall mixture effect and identifying influential components, particularly when associations are predominantly in the same direction. Quantile g-computation allows components to contribute to opposing directions and is therefore suitable for mixtures with heterogeneous effects. BKMR provides greater flexibility for identifying non-linear exposure–response relationships and interactions, although with greater computational complexity. Thus, model selection should be guided by research questions, mixture of characteristics, and study population.

For populations living in polluted areas, CV risk assessment should also consider exposure intensity, duration, environmental pathways, and individual susceptibility. A stratified approach combining environmental exposure assessment with exposure and effect biomarkers may help identify highly exposed or susceptible subgroups and support targeted biomonitoring and public health surveillance.

In several investigations, OS biomarkers, particularly F2-IsoPs and related lipid peroxidation products, as well as inflammatory biomarkers such as CRP, have been identified as potential mediators of these mixture effects.

The exposome framework further expands this perspective by considering TMMs as one component of the broader environmental exposure profile throughout the life course. TMMs often coexist with other CV risk factors, including particulate air pollution, persistent organic pollutants, noise, psychosocial stressors, and adverse socioeconomic conditions. These co-exposures may converge on common biological pathways, including OS, chronic inflammation, endothelial dysfunction, mitochondrial impairment, and epigenetic modifications, ultimately contributing to CVD development [93,94].

To translate these mixture insights into targeted public health interventions, future research in heavily contaminated regions should adopt stratified exposure assessment frameworks. Populations could be stratified along two complementary dimensions: (1) environmental exposure pathways including proximity to industrial point sources and contamination levels in drinking water, soil, food, and ambient environments, and (2) biological and socio-demographic susceptibility profiles including age, pre-existing cardiometabolic disease, nutritional deficiencies and, where feasible, relevant genetic susceptibility factors. Tiered biomonitoring in environmental hotspots could then be used to identify highly exposed or vulnerable subgroups and inform targeted CV risk reduction and environmental health interventions.

Future studies should therefore prioritize integrated exposomic approaches capable of simultaneously assessing multiple environmental stressors together with biological response markers. Such strategies, combined with advanced statistical methods and multi-omics technologies, may improve our understanding of the complex biological interactions underlying environmental CV risk and facilitate the identification of more robust biomarker signatures for early risk assessment [95].

The increasing recognition of complex environmental mixtures has also stimulated the search for more sensitive biomarkers capable of capturing the biological response to cumulative exposure. In this context, emerging molecular biomarkers and multi-omics approaches are expected to play a pivotal role in advancing environmental CV research.

### 4.5. Emerging Biomarkers and Future Directions

Although traditional biomarkers of OS and inflammation remain the most extensively investigated markers of the biological effect of TMM-induced CV toxicity, recent advances in molecular epidemiology are expanding the spectrum of candidate biomarkers capable of capturing early biological responses to environmental exposure. Emerging approaches integrating epigenetics, transcriptomics, metabolomics, proteomics, and extracellular vesicle analysis offer new opportunities to improve mechanistic understanding and CV risk assessment [96].

Among epigenetic biomarkers, alterations in DNA methylation patterns have consistently been associated with exposure to toxic metals, including As, Cd, Pb, and Hg. These epigenetic modifications may regulate the expression of genes involved in OS responses, inflammatory pathways, endothelial function, and lipid metabolism, potentially representing stable molecular signatures of cumulative exposure. Likewise, accelerated epigenetic aging, assessed through DNA methylation-based epigenetic clocks, has recently emerged as a promising indicator of biological aging and CV vulnerability in environmentally exposed populations [97].

Circulating microRNAs have also attracted considerable attention as sensitive regulators of CV homeostasis. Several microRNAs involved in endothelial dysfunction, vascular inflammation, myocardial remodeling, and OS have been reported to be dysregulated following TMMs exposure, suggesting their potential role as early biomarkers of subclinical CV injury [98,99]. Similarly, extracellular vesicles, including exosomes and microvesicles, are increasingly recognized as mediators of intercellular communication and may reflect endothelial activation, platelet activation, and inflammatory responses induced by environmental contaminants [100].

In parallel, untargeted metabolomic and proteomic approaches provide comprehensive characterization of biological perturbations associated with metal exposure. These high-throughput technologies enable the identification of metabolic pathways involved in mitochondrial dysfunction, oxidative damage, lipid peroxidation, and immune activation, offering the possibility of discovering novel biomarker panels with greater sensitivity and specificity than traditional individual biomarkers [101].

To successfully translate these emerging multi-group biomarkers into population studies and public health surveillance, their practical feasibility, analytical costs, and scalability must be carefully considered in comparison with traditional markers. Conventional biomarkers of OS and inflammation benefit from standardized, relatively low-cost, and high-throughput assays (e.g., ELISA, routine immunoassays), making them highly feasible for large-scale cohorts, although their exposure specificity may be limited. Emerging molecular approaches present distinct trade-offs. Epigenetic markers and microRNAs generally require more specialized analytical platforms and bioinformatic processing, although their stability in biobanked samples supports their application in population studies. Extracellular vesicle profiling provides detailed mechanistic insights but remains limited by relatively low throughput, high costs, and unstandardized isolation protocols. Likewise, untargeted metabolomics and proteomics involve higher per-sample costs, stringent sample handling requirements, and complex bioinformatic pipelines, which may limit their application in very large cohorts. Targeted molecular panels may therefore represent a more practical compromise between biological information and scalability. Overall, a tiered strategy, combining low-cost, high-throughput screening with targeted multi-omics profiling in selected high-risk subgroups, may offer a feasible approach for scalable environmental CV risk assessment.

Despite their considerable potential, most emerging biomarkers remain at an early stage of validation. Current evidence is largely derived from cross-sectional studies with relatively small sample sizes, and standardization of analytical methods is still limited. Future prospective studies integrating traditional biomarkers with multi-omics technologies and advanced exposure assessment within an exposome framework may facilitate the identification of robust biomarker signatures capable of improving early CV risk stratification and supporting precision environmental health strategies.

### 4.6. Strengths and Limitations of the Current Evidence

Several limitations should be considered when interpreting the findings of this review. Most included studies were cross-sectional or case–control in design, limiting the ability to establish temporal relationships and causal inference between TMMs exposure, biomarker alterations, and CVD development. Therefore, although OS and inflammatory biomarkers were frequently associated with both exposure and CVD outcomes, their utility as predictors of future CVDs remains uncertain.

In addition, substantial heterogeneity was observed across studies in terms of exposure assessment methods, biological matrices, biomarker selection, analytical approaches, and population characteristics. Many biomarkers, particularly CRP and cytokines, are influenced by multiple lifestyle and clinical factors, raising the possibility of residual confounding. Furthermore, most studies evaluated single metals, whereas real-world exposure typically involves complex mixtures, and relatively few investigations simultaneously assessed OS, inflammatory, and endothelial biomarkers.

Importantly, most included studies relied on single-time-point biomarker measurements, limiting the assessment of temporal dynamics and short-term exposure fluctuations. Pre-analytical and biological sources of variability were also rarely standardized or adequately controlled across studies. Factors such as diurnal variation (e.g., circadian fluctuations in OS and inflammatory markers), pre-sampling dietary intake (e.g., consumption of antioxidant-rich foods or dietary sources of trace metals such as seafood), and sample storage conditions (e.g., storage temperature, storage duration, and repeated freeze–thaw cycles) may affect biomarker concentrations and contribute to cross-study discrepancies. These methodological differences should therefore be considered when interpreting the heterogeneity of biomarker findings across studies.

To reduce such heterogeneity, future research should adopt standardized biological sample collection and analytical protocols, including harmonized sampling timeframes (e.g., standardized morning fasting blood collection or first-morning void urine collection), clearly defined pre-sampling dietary and fasting conditions, standardized sample pre-treatment and processing procedures, and controlled sub-zero storage conditions. Where feasible, the use of harmonized analytical platforms or the same validated reagent kits should also be considered. Detailed reporting of sampling time, dietary or fasting status, sample handling, storage temperature and duration, freeze–thaw history, and assay characteristics would further improve reproducibility and facilitate cross-study comparisons.

Despite these limitations, this review integrates evidence from environmental, occupational, and clinical studies and provides a comprehensive overview of mechanistic biomarkers linking TMMs exposure to CV toxicity, highlighting important gaps for future prospective research.

## 5. Conclusions

This narrative review highlights consistent epidemiological evidence linking environmental TMMs exposure to adverse CV outcomes through interconnected OS and inflammatory pathways. Among the biomarkers evaluated, lipid peroxidation markers, antioxidant defense measures, and inflammatory biomarkers, particularly CRP and hs-CRP, showed the most consistent associations with both TMMs exposure and markers of CV effects. Additional biomarkers, including adhesion molecules, cytokines, suPAR, and growth differentiation factor-15, further suggest that TMMs exposure induces a complex biological response extending beyond systemic inflammation alone.

However, interpretation of these findings is limited by the predominance of cross-sectional study designs, heterogeneity in exposure assessment and biomarker measurement, and variability across populations. These limitations preclude definitive conclusions regarding causality and predictive validity. Future research should prioritize large-scale prospective studies with repeated exposure assessment, standardized biomarker quantification, and comprehensive control of confounding factors. The integration of mixture-based exposure models, exposomics, and multi-omics approaches may help identify robust biomarker signatures reflecting cumulative exposure and CV risk.

Ultimately, a multimarker approach integrating OS, inflammatory, endothelial, and coagulation biomarkers may provide a more comprehensive framework for characterizing the biological effects of TMMs exposure and could contribute to future CV risk stratification. Furthermore, risk assessment and public health monitoring should be differentiated according to the exposure scenario rather than applying a uniform framework to occupational and environmental exposures.

For occupational exposure, where exposure intensity may be relatively high, and exposure sources and routes are more readily identifiable, risk assessment should prioritize workplace-specific exposure characterization and periodic biological monitoring. In addition to conventional exposure measurements, sensitive OS and inflammatory markers could potentially be incorporated as complementary mediators of early biological responses. Such biomonitoring may help identify workers with elevated biological responses, support exposure-control measures and occupational health surveillance, and provide evidence for optimizing engineering controls and personal protective measures. However, biomarker-based biological action thresholds should only be established after adequate prospective validation.

For the general population, environmental metal exposure is typically lower in intensity, chronic, and derived from multiple sources and pathways. Accordingly, public health assessment should emphasize population-level environmental biomonitoring, cumulative mixture-risk assessment, and surveillance of vulnerable subgroups, rather than relying primarily on individual biomarker measurements. Attention should be given to populations potentially more susceptible to CV effects, such as older adults and individuals with existing cardiometabolic risk factors. Where feasible, conventional biomarkers suitable for low-cost, high-throughput testing could support large-scale surveillance, whereas more specialized multimarker or multi-omics approaches could be applied to targeted high-risk or representative sub-cohorts. Thus, we propose a differentiated framework in which occupational settings emphasize source-specific exposure assessment and individual biological monitoring, whereas environmental settings emphasize population-level surveillance, cumulative risk assessment, and protection of susceptible subgroups. This stratified approach may improve the practical application of biomarker-based CV risk assessment while avoiding premature clinical or regulatory use. However, robust prospective validation is still required before these biomarkers can be incorporated into routine CV risk assessment or environmental health surveillance.

## Figures and Tables

**Table 1:** Summary included studies evaluating associations between toxic metals and metalloids exposure, oxidative stress (OS) biomarkers, and cardiovascular (CV) outcomes.

Study, Year, Country [Refs.]	Study Design/Sample Size/Data Source/Time	Matrix/Exposure Assessment	Matrix/Biomarkers	Analytical Method	Outcome Assessed	Main Results
Chen et al., 2025, China [36]	Longitudinal cohort, 45 healthy young adults, population-based, 2017–2018, 4 follow-up visits	Urine mixed-metals/ICP-MS	Urinary 8-OHdG, 8-iso-PGF2α	ELISA	Blood pressure	Cr, Mn, and Mo were positively associated with BP and 8-iso-PGF2α. 8-iso-PGF2α mediated associations. No association with 8-OHdG
Parvez et al., 2024, Bangladesh [37]	Cross-sectional, 199 e-waste workers, 104 controls, occupational, 2020–2022	Blood Pb, Cd/GFAAS; hair Hg/analyser	Urinary 8-OHdG	ELISA	Blood pressure	Pb significantly associated with increased systolic BP. Cd not associated. 8-OHdG was lower in exposed workers and did not mediate associations
Mandal et al., 2022, India [38]	Cross-sectional case–control, 110 MI cases, 110 controls, hospital-based, 2016–2017	Hair multi-metals/WD-XRF	Serum 8-OHdG	ELISA	MI	MI patients had higher Pb and As. Pb and As positively correlated with 8-OHdG. Cu and Mn were inversely associated with oxidative DNA damage
Kaur et al., 2022, India [39]	Case–control, 210 hypertensive, 210 controls, community-based, n.s.	Hair/nail Cd, As, Pb/ICP-MS	Serum TBARS, protein carbonyls, SOD, GSH, TAC	Spectrophotometry	Hypertension	Higher metals in hypertensive subjects. Increased TBARS, carbonyls, and decreased SOD, GSH, TAC
Wang et al., 2022, China [40]	Cross-sectional, 62 exposed to e-waste, 51 controls, e-waste exposed communities, 2017	Blood seven metals/ICP-MS	Blood MDA, 8-isoprostane	ELISA	Coronary heart disease	Co, Ni, and Pb positively associated with CHD biomarker. Ni strongly correlated with MDA and 8-isoprostane. No associations in controls
Yadav et al., 2022, India [41]	Cross-sectional comparative, 76 male exposed battery workers, 30 controls, occupational, n.s.	Blood Pb/ICP-MS	Blood MDA, total thiols, GSH, GST	TBARS, spectrophotometry	Blood pressure/cardiopulmonary risk	Higher CVD prevalence in exposed workers. Pb increased MDA and decreased antioxidant markers (thiols, GSH, GST)
Chung et al., 2020, Taiwan [42]	Cross-sectional, 965 residents near EAF, community-based, 2010–2016	Blood multi-metals/ICP-MS	Plasma MDA, GSH	TBARS, spectrophotometry	Blood pressure	Pb associated with increased BP and dyslipidaemia. Increased MDA and reduced GSH observed with exposure
Qu et al., 2019, China [43]	Cross-sectional, 144 exposed workers, 94 controls, occupational, n.s.	Blood Pb/GFAAS	Serum Total SOD, MDA	SOD kit, TBARS	Blood pressure, ECG abnormalities	Pb-exposed group had higher MDA and lower SOD activity than controls
Farzan et al., 2017, USA [44]	Cross-sectional, 418 participants, NHHS data, 2008–2012	Toenail and urine As/ICP-MS	Urinary 15-F2t-IsoP	ELISA	CVD risk	Urinary As positively associated with 15-F2t-IsoP. Better As metabolism linked to lower OS
Ko et al., 2017, Taiwan [45]	Cross-sectional, 117 welders, 49 controls, occupational, n.s.	Blood/urine multi metals/ICP-MS	Serum MDA	Fluorescence spectrophotometry	CVD risk	Welders had higher Cr and Mn. MDA positively correlated with Cr and Mn exposure

Abbreviations: 8-OHdG, 8-hydroxy-2′-deoxyguanosine; 8-iso-PGF2α, 8-iso-prostaglandin F2α; As, arsenic; BP, blood pressure; Cd, cadmium; CHD, coronary heart disease; Co, cobalt, Cr, chromium; CVD, cardiovascular disease; ELISA, enzyme-linked immunosorbent assay; EAF, electric arc furnace; GFAAS, graphite furnace atomic absorption spectrometry; GSH, glutathione; GST, glutathione S-transferase; Hg, mercury; ICP-MS, inductively coupled plasma mass spectrometry; MDA, malondialdehyde; MI, myocardial infarction; Mn, manganese; NHHS, New Hampshire Health Study; Ni, nickel; n.s., not specified; Pb, lead; SOD, superoxide dismutase; TAC, total antioxidant capacity; TBARS, thiobarbituric acid reactive substances; WD-XRF, wavelength dispersive X-ray fluorescence.

**Table 2:** Summary included studies evaluating associations between toxic metals and metalloids exposure, inflammatory biomarkers, and cardiovascular (CV) outcomes.

Study, Year, Country [Refs.]	Study Design/Sample Size/Data Source/Time	Matrix/Exposure Assessment	Matrix/Biomarker(s)	Analytical Method	Outcome Assessed	Main Results
Akinbode et al., 2025, USA [49]	Cross-sectional, 136 participants; All of US Research Program, 2018–2019	Blood Pb, As, Cd/standard laboratory procedures	Serum CRP	Standardized clinical laboratory immunoassay	Systolic blood pressure	Cd positively associated with CRP; Hg identified as strong predictor of SBP in mixture models
Chen et al., 2025, China [36]	Longitudinal cohort, 45 healthy young adults, 2017–2018, 4 follow-up visits	Urine mixed-metals/ICP-MS	Serum CRP, TGF-β1, ET-1, and VEGF	ELISA	Blood pressure	No significant association between metals and inflammation biomarkers
Ekholm et al., 2025, Sweden [50]	Cross-sectional, 2290 participants (subcohort 882), community-based 2014–2016	Blood and urine As, Cd, Pb/ICP-MS	40 inflammatory proteins (cytokines, chemokines, growth factors)	ECLIA	CVD and stroke	Long-term residence near glassworks associated with higher CVD/stroke prevalence and elevated inflammatory proteins as MCP-1, IL-1α, VEGF
Fang et al., 2025, USA [51]	Prospective Cohort, 40,687 participants, NHANES data, 1999–2018	Blood and urine metals/ICP-MS	Blood CRP	ELISA	CVD mortality	Pb, Cd, Hg associated with increased CVD mortality; CRP partially mediated effects
Sun et al., 2025, China [52]	Cross-sectional, 195 participants, 94 AMI patients, 101 controls, hospital-based, 2015–2020	Serum multi-elements/ICP-MS	Blood hs-CRP	Immunoturbidimetric clinical immunoassay	AMI	Cu positively associated with AMI prevalence/severity; hs-CRP partially mediated associations
Regencia et al., 2024, Philippines [53]	Within-subject repeated measures; 158 traffic enforcers, MMDA study	Blood and toenail Pb/AAS	Blood hs-CRP, sICAM-1, sVCAM-1	hs-CRP: immunoturbidimetric assay; sICAM-1 and sVCAM-1: ELISA	CV risk markers	Blood Pb associated with increased hs-CRP; no association with sICAM-1 or sVCAM-1
Xu et al., 2024, China [54]	Cross-sectional, 449 river vs. 551 non-river exposure, community-based, n.s.	Urinary 25 metals/ICP-MS	Serum ICAM-1, VCAM-1	ELISA	CHD risk	Higher Cu and Cd associated with increased VCAM-1 and higher CHD risk scores
Badeenezhad et al., 2023, Iran [55]	Repeated-measures panel study; 44 workers, community-based, 2020	Blood metals from PM10/ICP-MS	Blood CRP, TNF-α, fibrinogen	CRP: immunoturbidimetric assay; fibrinogen: Clauss method; TNF-α: ELISA	Blood pressure	Short-term exposure to As, Cd, Cr, showed a significant increase in BP, fibrinogen, and TNF levels and a decreased in CRP
Kim et al., 2023, Korea [56]	Cross-sectional study, 3773 adults, KNHANES data, 2016–2017	Blood Hg/AAS	Serum hs-CRP	Standard immunoturbidimetric assay	CVD risk	Hg positively associated with hs-CRP and elevated CVD risk profile
Sagha et al., 2023, Iran [57]	Case–control, 164 patients with MI and 61controls, hospital-based, n.s.	Serum As/FAAS	Serum hs-CRP	Automated immunoturbidimetric assay	MI	As exposure positively associated with MI severity and hs-CRP
Zheng et al., 2023, USA [58]	Cross-sectional, 9326 participants, NHANES data, 1999–2010	Urine metals/ICP-MS	Blood CRP	ELISA	Stroke	Cd associated with stroke risk; CRP mediated ~10% of association
de Andrade Freire et al., 2022, Brazil [59]	Cross sectional, 36 HF patients and 44 non- HF, hospital-based	Serum Cu, Zn/ICP-MS	Blood hs-CRP	Immunoturbidimetric high-sensitivity assay	Hearth Faliure	An increased Cu-to-Zn ratio in heart failure patients is associated with elevated hs-CRP levels
García-Esquinas et al., 2022, Spain [60]	Cross-sectional, 1942 elderly non-smokers, Seniors-ENRICA-2 data, 2015–2017	Blood Cd/ICP-MS	Serum IL-6, hs-CRP, GDF-15	GDF-15: ECLIA; hs-CRP and IL-6: standard immunoassays	CVD risk	Cd associated with inflammatory markers and increased CVD risk
Kunutsor et al., 2022, Finland [61]	Prospective cohort, 1866 participants, KIHD study, 1998–2001, 26.5 years median follow-up	Blood Cu, Zn/AAS	Serum hs-CRP	Immunometric assay	Heart failure	Elevated Cu/Zn ratio was associated with an increased risk of HF. Serum Cu/Zn ratio was positively correlated with hs-CRP
Ma et al., 2022, USA [62]	Cross-sectional, 38,223 participants, NHANES data, 1999–2016	Serum Cd/ICP-MS	BloodCRP	Automated immunoturbidimetric assay	CVD	Cd positively associated with CRP and multiple CVD outcomes
Mandal et al., 2022, India [38]	Cross-sectional case–control, 110 MI cases, 110 controls, hospital-based, 2016–2017	Hair multi-metals/WD-XR	Serum hs-CRP	Immunoturbidimetry	MI	Pb and As positively correlated with hs-CRP; Cu and Mn negatively correlated.
Yen et al., 2022, Taiwan [63]	Cross-sectional, 33 AIS patients, hospital-based, 2011–2013	Serum and urine As, Cd, Pb, Hg/ICP-MS	Blood CRP	Blood chemistry tests not specified	Acute Ischemic Stroke	In AIS levels of Pb, Cd, As, and Hg compared with controls. Urinary Hg and As were positively correlated with CRP
Andersson et al., 2021, Sweden [64]	Occupational longitudinal study (repeated measures), 72 workers, 2017–2018	Blood and urine Co/ICP-MS	Plasma and serum IL-6, IL-8, IL-10, TNF-α, SAA, hs-CRP	ELISA	CVD biomarkers	Co exposure was associated with increased IL-6 and weaker associations with TNF-α, IL-8, IL-10, and CC16
Nguyen et al., 2021, Korea [65]	Cross-sectional, 9, 602 participants, KNHANES data, 2009–2103, 2016–2019	Blood Cd, Pb, Hg/GFAAS and Hg analyser	Serum hs-CRP	Immunoturbidimetry	10-year CVD risk	Metals positively associated with hs-CRP and CVD risk score
Chung et al., 2020, Taiwan [42]	Cross-sectional, 965 residents near EAF, community-based, 2010–2016	Blood multi-metals/ICP-MS	Plasma IL-6, IL-8	Fluorescent multiplexed bead-based immunoassay	Blood pressure	Blood Pb levels were significantly associated with increased BP. No clear associations with inflammatory markers
Obeng-Gyasi et al., 2020, USA [66]	Cross-sectional, 11, 145 participants, NHANES data, 2007–2010	Urine Cd/ICP-MS	Blood CRP	Nephelometer	CVD	Chronic Cd exposure is associated with increased CRP
Sobel et al., 2020, USA [67]	Cross-sectional, 6814 participants, MESA data, 2000–2002	Urinary As/ICP-MS	Blood IL-6, CRP, fibrinogen, ICAM-1	ELISA	Subclinical CVD	No significant associations between As and inflammatory biomarkers
Yuan et al., 2020, China [68]	Cross-sectional, 2282 participants, population- based, 2008–2010	Plasma multi-metals/ICP-MS	Serum CRP	Latex turbidimetric immunoassay	CHD	CRP positively associated only with Cu
Darroudi et al., 2019, Iran [69]	Cross-sectional, 9558 participants, MASHAD data, 2010–2011	Serum Cu, Zn/FAAS	Blood hs-CRP	Routine laboratory methods	Hypertension	Higher Cu associated with increased hs-CRP and hypertension risk
Palaniswamy et al., 2019 Finland [70]	Cross-sectional, 206 healthy participants, NFBC1966 data	Blood Cu/ICP-MS	Blood hs-CRP, activePAI-1	Hs-CRP: ELISA; PAI-1: multiplex array	CVD	Cu was found positively associated with AGP and hs-CRP. No association for active PAI-1 was found
Camaj et al., 2018, Yugoslavia [71]	Prospective cohort, 101 participants, population- based, follow-up in 2011	Blood Pb/GFAAS	Serum ICAM-1, VCAM-1	ELISA	Blood pressure	Pb associated with endothelial dysfunction and increased BP
Han et al., 2018, China [72]	Case–control; 85 ICH patients, 12 hypertension controls, 32 healthy controls, hospital-based, n.s.	Serum Cu/GFAAS	Serum hs-CRP	Immunonephelometric assay	Intracerebral hemorrhage	Elevated Cu associated with worse outcomes and higher inflammation
Ngala et al., 2018, Ghana [73]	Case–control, 163 diabetes patients and 168 controls, hospital-based, 2014–2015	Plasma Cr/AAS	Serum hs-CRP	ELISA	Hypertension	Cr deficiency associated with hypertension and elevated CRP
Obeng-Gyasi et al., 2018 USA [74]	Cross-sectional, 12,153 participants, NHANES data, 2007–2010	Blood Pb/ICP-MS	Serum Hs-CRP	Latex-enhanced immunonephelometric assay	CVD	Findings reported associations between Pb exposure, elevated hs-CRP, and increased BP
Fagerberg et al., 2017 Sweden [75]	Cross-sectional, 4648 participants, MDCS data, 1991–1994	Blood Cd/ICP-MS	Plasma suPAR, CRP	ELISA	Atherosclerotic disease	Cd exposure was associated with suPAR but not with CRP
Farzan et al., 2017, USA [44]	Cross-sectional, 418 participants; NHHS data; 2008–2012	Toenail and urine As/ICP-MS	Plasma inflammation VCAM-1 MMP-9, TNF−α and ICAM-1	Multiplex bead-based immunoassay platform	CVD risk	As was positively associated with VCAM-1, and non-significant with ICAM-1
Moon et al., 2017, USA [76]	Cross-sectional, 2700/1984 participants, SHS data, 1989–1999	Urine As/HPLC coupled to ICP-MS,	Plasma PAI-1, fibrinogen, hs-CRP 2	ELISA	CVD mortality	Pb associated with CVD mortality independent of CRP
Prokopowicz et al., 2017, Poland [77]	Cross-sectional, 231 Pb-exposed workers, occupational, n.s.	Blood Pb/AAS	Serum Hs-CRP, Fb, Hcy, ADMA.	Hs-CRP: turbidimetric, Fb: using standard Clauss method.	Atherosclerotic disease	Positive association between blood Pb levels and blood pressure. Pb concentrations correlated with all biomarkers except for ADMA
Skalny et al., 2017, Russia [78]	Case–control, 30 cases with AIS and 30 health controls, hospital-based, n.s.	Serum multi-metals/ICP-MS	Serum C4, C3a, VEGF, S100B protein, NR2Ab, and TAS, plasma fibrinogen, D-dimer	C3a, C4: turbidimetric, TAS: immunodiagnostic, Fibrinogen, D-dimer: ELISA	Acute ischemic stroke	Elevated Cu, Mn, As, Pb, and Ni were associated with fibrinogen, D-dimer, complement factors, and VEGF
Aoki et al., 2016, USA [79]	Cohort prospective, 18,602 participants, NHANES data, 1999–2010	Blood Pb/ICP-MS	Serum CRP	LETIA	CVD mortality	Pb-CVD mortality association was not mediated through CRP
Barregard et al., 2016, Sweden [80]	Prospective cohort, 4819 participants; MDCS data, 17-year follow-up	Blood Cd/ICP-MS	Blood hs-CRP	Standard methods	CVD	Cd associated with increased hs-CRP and CVD risk

Abbreviations: AAS, atomic absorption spectrometry; ADMA, Asymmetric Dimethylarginine; AMI, acute myocardial infarction; As, arsenic; BP, blood pressure; Cd, cadmium; CHD, coronary heart disease; Co, cobalt; Cr, chromium; CRP, C-reactive protein; Cu, copper; CV, cardiovascular; CVD, cardiovascular disease; ECLIA, electrochemiluminescence immunoassay; ELISA, enzyme-linked immunosorbent assay; ET-1, endothelin-1; FAAS, flame atomic absorption spectrometry; GDF-15, growth differentiation factor-15; GFAAS, graphite furnace atomic absorption spectrometry; HF, heart failure; hs-CRP, high-sensitivity C-reactive protein; Hg, mercury; HPLC, High-Performance Liquid Chromatography; ICAM-1, intercellular adhesion molecule-1; ICH, intracerebral hemorrhage; ICP-MS, inductively coupled plasma mass spectrometry; IL, interleukin; IL-1α, interleukin-1 alpha; IL-6, interleukin-6; IL-8, interleukin-8; IL-10, interleukin-10; KIHD, Kuopio Ischemic Heart Disease Risk Factor Study; KNHANES, Korea National Health and Nutrition Examination Survey; MASHAD, Mashhad Stroke and Heart Atherosclerotic Disorder Study; MCP-1, monocyte chemoattractant protein-1; MDCS, Malmö Diet and Cancer Study; MESA, Multi-Ethnic Study of Atherosclerosis; MI, myocardial infarction; Mn, manganese; NFBC; Northern Finland Birth Cohort; NHANES, National Health and Nutrition Examination Survey; NHHS, New Hampshire Health Study; Ni, nickel; n.s., not specified; Pb, lead; SAA, serum amyloid A; SBP, systolic blood pressure; suPAR, soluble urokinase plasminogen activator receptor; TNF-α, tumor necrosis factor alpha; VCAM-1, vascular cell adhesion molecule-1; sVCAM-1, soluble vascular cell adhesion molecule-1; VEGF, vascular endothelial growth factor; Zn, zinc; LETIA, Latex-enhanced nephelometry immunoassay.

## Data Availability

No new data was created or analyzed in this study. Data sharing is not applicable to this article.

## Abbreviations

The following abbreviations are used in this manuscript:

As	Arsenic
BKMR	Bayesian kernel machine regression
Cd	Cadmium
Co	Cobalt
CV	Cardiovascular
Cr	Chromium
CRP	C-Reactive Protein
Cu	Copper
CVD/CVDs	Cardiovascular Disease/s
F2-IsoPs	F2-isoprostanes
GSH	glutathione
Hg	Mercury
hs-CRP	high-sensitivity C-Reactive Protein
ICAM-1	Intercellular adhesion molecule-1
sICAM-1	Soluble intercellular adhesion molecule-1
IL	Interleukin
KNHANES	Korean National Health and Nutrition Examination Survey
MCP-1	Monocyte chemoattract-ant protein-1
MDA	Malondialdehyde
MI	Myocardial Infarction.
Mn	Manganese
NF-κB	nuclear factor-κ-gene binding
NHANES	National Health and Nutrition Examination Survey
NLRP3	nucleotide-binding oligomerization domain-like receptor containing pyrin domain 3
Nrf2	nuclear factor erythroid 2-related factor 2
OS	Oxidative stress
PAI-1	Plasminogen Activator Inhibitor-1
Pb	Lead
ROS	Reactive Oxygen Species
SOD	superoxide dismutase
suPAR	Urokinase Plasminogen Activator Receptor
TBARs	Thiobarbituric acid reactive substances
TMMs	Toxic metals and metalloids
TNF-α	Tumor necrosis factor-alpha
VCAM-1	Vascular cell adhesion molecule-1
sVCAM-1	Soluble vascular cell adhesion molecule-1
VEGF	vascular endothelial growth factor
WQS	Weighted Quantile Sum
Zn	Zinc
8-iso-PGF2α	8-iso-prostaglandin F2α
8-OHdG	8-hydroxy-2′-deoxyguanosine
